# Retinal and anterior eye compartments derive from a common progenitor pool in the avian optic cup

**Published:** 2011-12-20

**Authors:** Sara J. Venters, Paulina D. Cuenca, Jeanette Hyer

**Affiliations:** Department of Ophthalmology, Department of Neurosurgery, University of California, San Francisco, CA

## Abstract

**Purpose:**

The optic cup is created through invagination of the optic vesicle. The morphogenetic rearrangement creates a double-layered cup, with a hinge (the Optic Cup Lip) where the epithelium bends back upon itself. Shortly after the optic cup forms, it is thought to be sub-divided into separate lineages: i) pigmented epithelium in the outer layer; ii) presumptive iris and ciliary body at the most anterior aspect of the inner layer; and iii) presumptive neural retina in the remainder of the inner layer. We test the native developmental potential of the anterior cup to determine if it normally contributes to the retina.

**Methods:**

Vital dye and green fluorescent protein (GFP) expressing replication-incompetent retroviral vectors were used to label cells in the nascent optic cup and follow their direct progeny throughout development. Label was applied to either the optic cup lip (n=40), or to the domain just posterior to the lip (n=20). Retroviral labeling is a permanent lineage marker and enabled the analysis of advanced stages of development.

**Results:**

Labeling within the optic cup gave rise to labeled progeny in the posterior optic cup that differentiated as neural retina (20 of 20). In contrast, labeling cells in the optic cup lip gave rise to progeny of labeled cells arrayed in a linear progression, from the lip into the neural retina (36 of 40). Label was retained in cells at the optic cup lip, regardless of age at examination. In older embryos, labeled progeny delaminated from the optic cup lip to differentiate as muscle of the pupillary margin.

**Conclusions:**

The data show that the cells at the optic cup lip are a common progenitor population for pigmented epithelium, anterior eye tissues (ciliary body, iris, and pupillary muscle) and retinal neurons. The findings are supportive of an interpretation where the optic cup lip is a specialized niche containing a multipotent progenitor population.

## Introduction

The eye develops from the embryonic neuroepithelium. The optic anlage is first identifiable as paired evaginations, called optic vesicles, off the anterior neural tube. As development proceeds, the distal optic vesicle invaginates, in concert with overt differentiation of the lens, forming the optic cup. The optic cup is a continuous epithelium comprising the inner and outer epithelial layers. The optic cup lip (OCL) is the hinge point where the epithelium bends, and marks the boundary between the inner/nonpigmented and outer/pigmented layers of the eye epithelium. With continuing development, the optic cup expands and differentiates into the multiple tissues of the eye. In the anterior of the eye, the optic epithelium is nonneural and matures as ciliary body and iris epithelia. Thus, the adult OCL is the iris edge that lines the pupil.

The proliferative populations of the anterior eye (ciliary body and iris epithelia) are the most promising for stem cell therapy, as these tissues are readily accessible. However, there is some controversy about the potential for this anterior epithelium to replace sensory retina cell types ([1-8], reviewed in [9]). In addition, it is unclear whether there is any anatomic basis for the observed retinal potential from cells derived from the ciliary body and iris, other than their embryological relationship to the retina.

Throughout studies of retinal development in higher vertebrates, there is a prevailing depiction of the neural retina and presumptive anterior eye tissue (the iris and ciliary body) segregating early in eye morphogenesis. As transcription factors such as *Otx1* (vertebrate homolog of the *Drosophila* orthodenticle homeobox gene) and *Msx1* (homolog of the *Drosophila* muscle segment homeobox gene), and functional proteins such as Collagen IX, are expressed soon after optic cup formation in both the chick and mouse eye, it has been assumed that the anterior portion of the cup, including the OCL, is already specified and does not contribute to the neural retina [8,10-14]. In addition, the available lineage analysis studies have indicated that the neural retina derives from the distal extreme of the optic vesicle [15,16]. Thus, the current understanding, based on gene expression studies, is that the anterior structures—the ciliary body and iris—are the sole derivatives of the anterior extreme of the newly formed optic cup.

We have previously shown that misexpression of *FGF4* (fibroblast growth factor 4) is sufficient to create an ectopic boundary between pigmented and nonpigmented tissue, and at the boundary, ectopic ciliary body tissue is induced [10]. Further, *Wnt2b* (vertebrate homolog of the *Drosophila* wingless gene) expression is induced at ectopic boundaries, mimicking its native OCL expression [17-20]. These observations have led us to conclude that the boundary between pigmented and nonpigmented tissues represents a distinct, undefined tissue state. However, any role for this boundary or the genes expressed therein has been difficult to dissect, as no specialized role for the native boundary, at the OCL, has yet been determined. We have employed direct lineage methods to analyze the potential of the OCL as compared to nonboundary optic cup tissue.

This study demonstrates that, in the avian eye, the neural retina, associated pigment epithelium (RPE), and all tissues (excluding mesenchymal derivatives) of the anterior eye derive from cells originally resident in the OCL. We show that the OCL is a pool of long-lived progenitors that contribute progeny to all tissues of the eye during the entirety of eye morphogenesis. Finally, we propose that the OCL is the source of peripheral retinal growth during development in higher vertebrates and that the anterior optic cup in chicks is not specialized for the production of ciliary body or iris, despite specific gene expression patterns.

## Methods

### Replication-incompetent retroviruses

Proviral plasmids used encode either GFP (green fluorescent protein) or membrane-bound TdTomato (a tandemized version of a red fluorescent protein varient developed by Dr. Robert Tsien of the University of California, San Deigo) co-expressed with nGFP (nuclear localized GFP) under the control of a spleen necrosis virus promoter [21,22]. The plasmid encoding membrane-bound TdTomato/nGFP was the generous gift of George Trichas and Shankar Srinivas [23]. nGFP allowed clearer marker visualization in pigmented cells. The XbaI fragment containing the fluorescent proteins was excised and cloned into the spleen necrosis virus retroviral backbone. Replication-incompetent retrovirus was prepared as described [22]. Briefly, the proviral plasmid and a plasmid containing the VSV-G (vesicular stomatitis virus) envelope protein were transfected using calcium phosphate into the Phoenix packaging cell line [24], which has stably incorporated the MLV (murine leukemia virus) *gag* and *pol* genes. The virus was concentrated from culture supernatant of six 10 cm culture dishes by ultracentrifugation at 50,000× g in a Beckman ultracentrifuge. Concentrated virus titer was determined using D17 cells (dog osteosarcoma cells; American Type Culture Collection (ATCC), Manassus VA). DMSO (dimethyl sulfoxide) was added to concentrated viral solution (final concentration 10%) before injection. For some injections, Fast Green (F7258; Sigma Chemical Co., St. Louis MO) was added to increase contrast (final concentration 0.01%).

### Injections

Fertilized chicken eggs (*Gallus gallus domesticus*) were obtained from a local breeder (Petaluma Farms, Petaluma CA). Eggs were incubated at 38 °C in a humid incubator until embryonic day (E) 3. For injection, the eggshell was carefully removed and the extra-embryonic membranes over the eye were cut. The OCL and optic cup were targeted from the outer layer: Nile blue sulfate, tungsten knives, and pancreatin were used to clear the surface ectoderm over the target site, exposing either the RPE (retinal pigmented epithelium) or OCL. Applying 1.5% Nile blue sulfate (N-5632; Sigma Chemical Co.) to the surface ectoderm facilitates removal. Pancreatin (915725-013; Gibco/Life Technologies, Grand Island NY) at a concentration of 0.25 mg/ml was applied with pulled glass capillaries to the opened ectodermal area. Excess enzyme was washed off with PBS. Concentrated virus supernatant or dye was backfilled into a pulled glass microcapillary pipette and applied to the eye using a pressure injector (model PLI-100; Harvard Apparatus, Holliston MA). DiI (1,1'-dioctadecyl-3,3,3'3'-tetramethylindocarbocyanine perchlorate, Molecular Probes, Portland OR) was diluted to 2 mg/ml in tetraglycol (T3396; Sigma Chemical Co.). Focal labeling was confirmed in DiI labeling as described below for assessment of labeling output. A pressure injector was used to deliver the smallest observable amount to the exposed tissue. Eggs were sealed with Parafilm (Pechiney Plastic Packaging, Chicago IL) and reincubated for 1–14 days until embryos reached between E4 and E17.

### Injection output

Output from labeled cells was assessed in live embryos using a Leica fluorescent stereomicroscope fitted with a DFC500 camera (Leica Microsystems, Buffalo Grove IL). Following recording, embryos were fixed in 2% paraformaldehyde and processed for immunohistochemistry. Eyes where no label was evident were dissected to examine the posterior neural retina and RPE.

### Immunohistochemistry

Embryo heads were sunk in PBS containing 20% sucrose, embedded in a 1:1 solution of 20% sucrose in PBS:OCT (Sakura Finetek, Torrance CA) and frozen on dry ice. Serial cryosections (10–12 µm) were collected and processed for immunohistochemistry [25] by 1) blocking in PBS:1% bovine serum album: 1% normal goat serum for 1 h; 2) incubating in primary antibody overnight and then secondary antibody for 1 h at the indicated dilutions into the blocking solution; and 3) mounting in DAPI (4',6-diamidino-2-phenylindole) containing mounting media (Vector Labs, Burlingame, CA). Primary antibodies used were mouse anti-Collagen IX clone 2C2 (Developmental Studies Hybridoma Bank [DSHB], Iowa City, IA), mouse anti-HuC (Molecular Probes, Portland, OR), rabbit anti-GFP (Rockland Immunochemicals, Gilbertsville PA), mouse anti–smooth muscle alpha-actin (Dako North America, Carpinteria CA), mouse anti-BrdU (DSHB), mouse anti-Pax6 (DSHB), mouse anti-Pax3 [26], mouse anti-MF20 (DSHB), mouse anti-Visinin (DSHB), and mouse anti-NaK-ATPase clone a5 (DSHB), diluted to 1:250, 1:100, 1:2000, 1:200, 1:250, 1:250, 1:100, 1:250, 1:200, and 1:400, respectively. Antibody labeling was visualized using complimentary secondary antibodies conjugated with either Alexa488 or 594 (Molecular Probes), diluted to 1:500. Images were collected and compiled with a Zeiss Axiophot microscope and Spot camera (Diagnostic Instruments, Sterling Heights MI), and Adobe Photoshop CS3 v10.0.1 (Adobe Microsystems Inc. San Jose CA). Wholemount immunohistochemistry was performed as described previously [22] on isolated tissues. Images were captured using a LeicaTCS SPEII confocal microscope (Leica Microsystems, Buffalo Grove IL) with Leica Application Suite Advance Fluorescence software version 2.4.1.

### Bromodeoxyuridine exposure and detection

Fifty microliters of 10 mM BrdU (bromodeoxyuridine) in PBS were injected into the yolk of E14 embryos and eggs reincubated for 4–8 h. Embryos were processed for sectioning and immunohistochemistry as described above. Sections for BrdU labeling were incubated in 1 M HCl at 37 °C for 15 min, followed by 0.1 M sodium borate washes before immunolabeling.

### Statistical analysis

Data were analyzed using SPSS (IBM, Armonk NY). The statistical significance of BrdU incorporation between the anterior 100 µm and posterior of the iris sphincter muscle was tested using an independent sample *t* test. In total, 6,282 nuclei were counted, encompassing three embryos at each time point.

## Results

### Optic cup lip cells produce daughters arrayed linearly through the optic cup tissue

**Figure 1:**
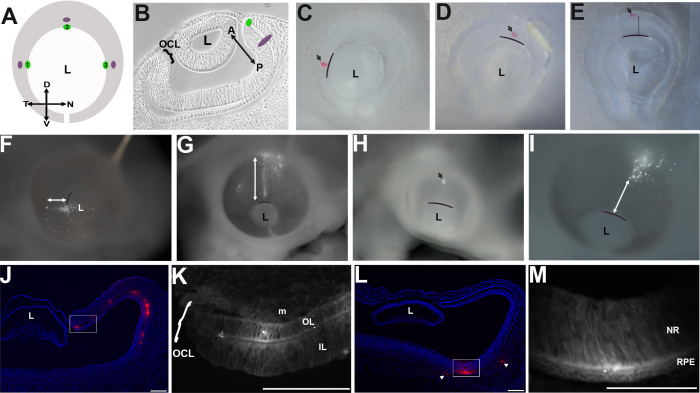
Optic cup labeling reveals a precise growth pattern for cells derived from the optic cup lip. **A**, **B**: Graphics of the labeling targets in the E3 optic cup, viewed as a wholemount (**A**) and transverse section (**B**). The crosshairs in **A** highlight orientation in all wholemount eyes: D-dorsal; V-ventral; N-nasal; T-temporal. In **B**, A-anterior (from lens), P-posterior (toward head) axis, specific for the eye, is indicated. The OCL (green dot) or optic cup (purple dot) was targeted around the circumference of the pupil. **C-M**: DiI labeled embryos: Embryos were imaged immediately after labeling (**C**-**E**) and following reincubation (**F**-**M**). The curved bars outline the boundary between the lens and optic cup. **C**, **D**: Dye targeted to the OCL (arrowhead). **E**: Dye targeted on the optic cup (arrowhead). **F**, **G**: OCL targeted embryos after 24 (**F**) and 48 (**G**) h reincubation. Labeled cells are present as a spoke-line extending from the OCL, adjacent to the lens, toward the posterior eye. The line highlights the extent of labeled cells. **H**, **I**: Resultant dye pattern 24 (**H**) and 48 (**I**) h after labeling the optic cup, as in **E**. Discrete patches of dye are found posterior to the OCL (arrow). The scattered dye is in the mesenchyme overlying the neuroepithelium. No linear labeling is found. **J**-**M**: Sections through the labeled tissue 48 h after targeting the OCL (**J**, **K**) or optic cup (**L**, **M**). **J**: After OCL labeling, the dye is retained in the OCL and is distributed through the anterior optic cup. **K**: A higher magnification of the boxed area in **J**. Dye is present in the inner and outer epithelial layers of the optic cup and in the overlying mesenchyme. **L**: After optic cup labeling, dye is found as a discrete patch in the posterior eye and is absent from the front of the eye. Some dye is present in the mesenchyme overlying the neuroepithelium (arrowheads). **M**: A higher magnification of the boxed area in **L**. Dye is present in both the neural retina and overlying retinal pigmented epithelium. Scale Bars: 100 µm. OCL; Optic Cup Lip, L; lens, NR; Neural retina, RPE; Retinal pigmented epithelium, CB; Ciliary body, IL; Inner layer, OL; Outer layer.

The OCL is a hinged epithelium throughout development. Similarly, hinged epithelial lips have been shown to act as progenitor pools during the morphogenesis of other embryonic tissues, e.g., the dorsomedial lip of the somite dermomyotome [27-29]. We hypothesized that the OCL likewise has a specialized role as a progenitor pool during eye morphogenesis. To test the potential of the optic cup to contribute to tissues of the anterior eye, DiI was targeted to either the OCL (Figure 1A,B; green dot) or to the optic cup, posterior to the OCL (Figure 1A,B; purple dot). For this study, the OCL and optic cup were targeted from the outer layer: Nile blue sulfate and pancreatin were used to clear the surface ectoderm over the target site, exposing either the RPE or OCL. A pressure injector was used to deliver the smallest observable amount of DiI to the exposed tissue. The injection site was immediately verified and recorded (Figure 1C-E), and the embryos reincubated as indicated.

Targeting the OCL cells consistently gave rise to labeled populations running from the OCL toward the posterior of the eye (Figure 1F,G and Table 1). Throughout the study, this pattern is called a spoke-line, to denote the center-to-edge pattern of the label, where the center is the lens. In contrast, labeling cells in a more posterior position resulted in a discrete patch of dye in the optic cup, situated back from the lens (Figure 1H-I and Table 1). Targeted labeling on the general optic cup resulted in discrete patches removed from the lens in all cases (20 embryos), while OCL labeling gave rise to spoke-lines of tagged cells in 36/40 embryos with the label retained in the OCL in 37/40 embryos. These labeling patterns were observed at all targeted positions circumferentially around the lens. Sections through dye-labeled eyes confirmed label through the anterior eye when the OCL was targeted (Figure 1J,K), and discrete label in the posterior neural retina and overlying RPE, with no label in the anterior compartment of the eye, when the general optic cup was targeted (Figure 1L,M).

**Table 1:** Summary of labeling the optic cup lip (OCL) or optic cup.

**Target**	**Total embryos**	**Age range (somite number)**	**Age range (HH stages)**	**Reincubation range (days)**	**Label retained in OCL**	**Radial**	**Discrete label**
OCL	40	18–33	12+ to 18	1–15	37/40 (92.5%)	36/40 (90%)	1/40*
Optic Cup	20	23–35		1.5–4	0/20	0/20	20/20

The majority of OCL embryos (32/40) were labeled at ≥HH14. Those embryos that had not retained label in the OCL at the end of the re-incubation period were HH15–16 at labeling. The summarized data are the result of injecting >500 embryos. All embryos with any GFP expression were scored, in any particular injection run typically 5% of embryos were infected. *1 embryo had GFP expression in a few cells immediately back from the OCL.

### The ciliary body and peripheral neural retina are derived from the optic cup lip

DiI and other lineage dyes cannot be followed clearly for extended periods in proliferative tissues due to dye dilution. Therefore, to follow labeled cells into differentiated tissues, we used a replication-incompetent retrovirus encoding GFP to infect OCL cells. This virus has unique elements, including the use of a strong avian promoter that allows the direct visualization of GFP fluorescence at single-cell resolution and no observed helper viruses [22]. As in the DiI injections, the virus was introduced from outside the optic cup. Nile blue sulfate and pancreatin were used to clear the surface ectoderm over the optic cup lip. The virus in these studies is pseudotyped with the VSV-G envelope protein, which uses a membrane fusion mechanism to enter the cell [30]. The viral solution also contains DMSO and Fast Green as a contrast stain. A blunted micropipette (with an internal diameter of 10 µm) was pressed against the OCL and the balance pressure of the pressure injector adjusted to allow the viral solution to contact the tissue, without flowing past the contact between needle and tissue. With this technique, the viral solution did not enter the intraocular space between the forming neural retina or RPE; nor did it enter the vitreal space.

GFP is only expressed in cells initially infected with the retrovirus and their direct progeny. As a permanent inherited marker, it is not selectively diluted out from highly proliferative populations, and thus allows accurate analysis of the scope of the target population output. The combination of a unique molecular reagent and a new surgical approach allowed us to specifically target and label small populations at the OCL, as confirmed with dye labeling. Following 3- to 4-day reincubation, embryos were removed from the egg and examined directly under a fluorescent dissecting microscope.

**Figure 2:**
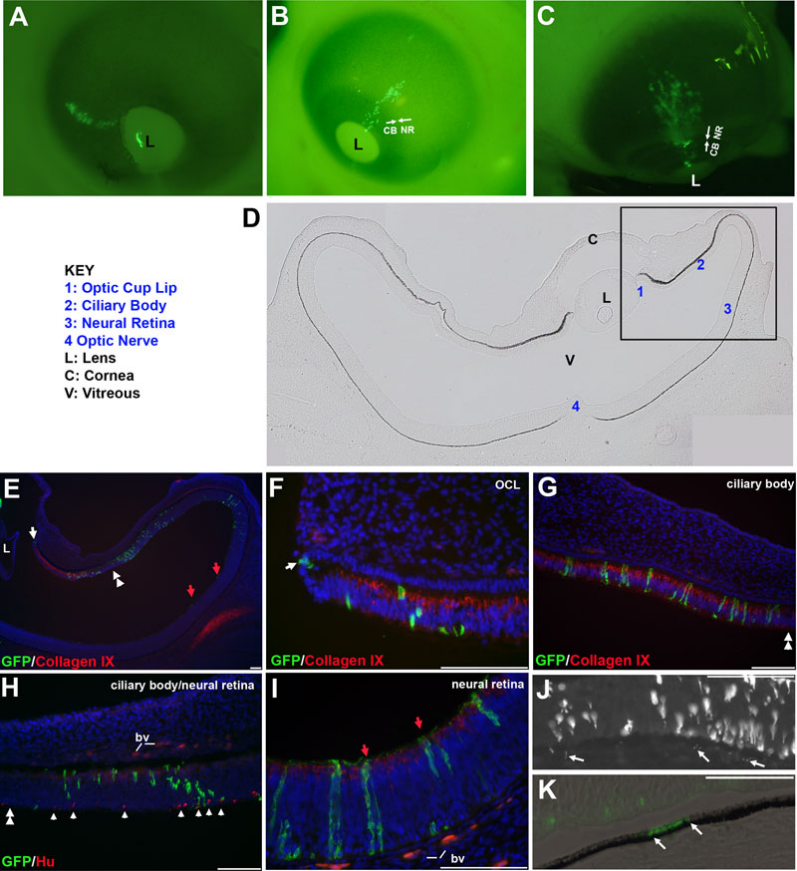
Cells derived from the OCL are distributed through the peripheral neural retina and ciliary body. **A**-**C**: Whole mount views of eyes infected with GFP-expressing replication-incompetent retrovirus targeted to the OCL. Embryos were reincubated until E4.5 (**A**), E6.5 (**B**), and E9 (**C**). **A**: GFP expressing cells are evident as a spoke-line emanating from the OCL adjacent to the lens. **B**, **C**: The GFP expressing cells traverse the morphological boundary between the ciliary body and the peripheral neural retina (arrows, **B** and **C). D**-**K**: Coronal sections through the GFP expressing region of an OCL targeted eye. **D**: Phase contrast image of the eye with relevant features indicated. Boxed area represented at higher magnification in panels **E**-**K**. **E**: GFP is evident at the OCL (arrow), in the Collagen IX expressing ciliary body (red signal), posterior to the Collagen IX zone in the optic epithelia and axons (red arrows). Double arrowheads indicate the border between ciliary body and neural retina. **F**: GFP expressing cells are located in the hinge at the OCL (arrow) and in the anterior Collagen IX expressing region. **G**: GFP expressing cells are seen throughout the presumptive ciliary body. **H**: GFP expressing cells are located at the posterior ciliary body/ peripheral NR, where Hu expression is evident in a few cells (arrowheads). Blood vessels (bv) are autofluorescent. **I**: GFP expressing cells are present in the Hu expressing neural retina and GFP labeled axons are apparent (red arrows). **J**: GFP is also present in the outer, pigmented epithelium (arrows) over the neural retina. **K**: Progeny of cells (different embryo) infected with a virus encoding nuclear localized GFP show abundant GFP expression in the pigmented epithelium overlying the neural retina (arrows). Scale Bars: 100 µm. The anterior of the eye is to the left. OCL; Optic Cup Lip, L; lens, NR; neural retina, CB; ciliary body, bv; blood vessels.

Wholemount views of eyes after targeting the OCL showed that GFP expression mirrored the spoke-like linear distribution seen with DiI (Figure 2A-C). Targeting around the circumference of the lens produced spoke-line patterns positioned circumferentially around the lens (Figure 2A versus Figure 2B). Labeled cells extended through the boundary between the presumptive ciliary body and peripheral neural retina (as indicated, Figure 2B).

To confirm the distribution of tagged cells, infected eyes were completely sectioned and the GFP distribution was analyzed (Figure 2E-K). GFP-expressing cells were located in the OCL (white arrow; Figure 2E,F) and extended evenly throughout the optic cup tissue, to the posterior retina (red arrows; Figure 2E,I). GFP+ cells were found in the presumptive ciliary body, identified by expression of Collagen IX (Figure 2G). Continuing posteriorly, GFP+ cells were present at the periphery of the neural retina (double white arrowhead; Figure E,G,H), between the Collagen IX+ and the neural retina domain, as determined by expression of the neuronal RNA–binding protein Hu (single arrowheads; Figure 2H) [31]. In the more posterior neural retina (Figure 2I), GFP+ columnar retina clones and labeled axonal projections were seen at the basal neural retina (Figure 2E,I; red arrows). GFP expression was apparent in the RPE overlying the GFP-expressing neural retina and ciliary body, but was faintly expressed in comparison with the expression in the inner epithelia of either tissue (Figure 2J; arrows). To confirm that this was an artifact resulting from RPE cell morphology (the ratio between cytoplasmic and nuclear volume) and interference from the pigment granules for visualizing cytoplasmic GFP, the OCL was similarly targeted with a retrovirus encoding a nuclear-localized GFP. Nuclear GFP expression confirmed that progeny from the OCL equally populated the outer, pigmented, and inner epithelia of these tissues (Figure 2K; arrows). OCL-derived cells gave rise to both neural retina and RPE regardless of the embryo age when the OCL was labeled (HH12+ to HH18, Table 1).

### Label applied to non–optic cup lip anterior populations has distinct progeny distribution patterns

**Figure 3:**
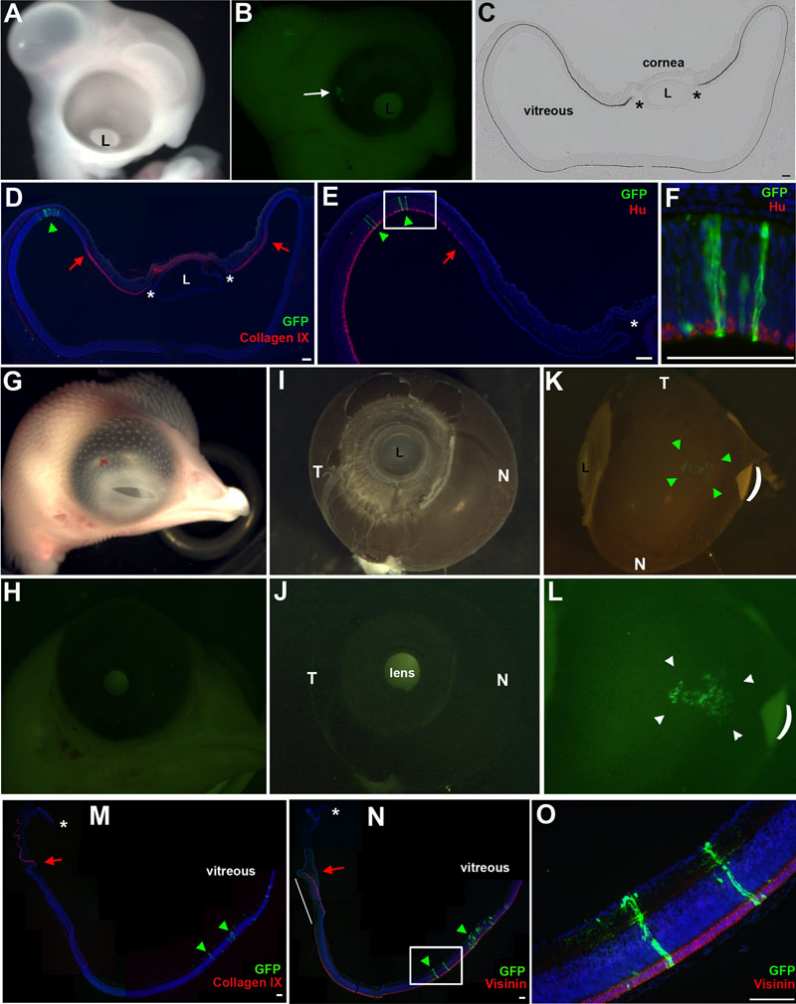
Non-optic cup lip derivatives populate the posterior neural retina. **A**-**E**: The optic cup was targeted with retrovirus, as described in Figure 1E, and reincubated until E5 (**A**). **B**: Whole mount view of GFP expressing cells (arrow), located at a distance from the OCL. **C**, **D**: Transverse sections through the GFP expressing region. GFP+ cells do not populate the ciliary body (Collagen IX signal) or the margin between the ciliary body and neural retina (red arrow). Asterisks indicate OCL. **E**, **F**: GFP+ cells are found only in the Hu+ neural retina. Boxed region in **E** is represented at higher magnification in **F**. **G**-**O**: Results of optic cup labeling, examined at E10. **G**, **H**: GFP+ cells are not immediately detectable in whole mount view of head. **I**, **J**: Infected eye dissected from head, nasal (N) and temporal (T) orientation as indicated. No GFP signal is seen in anterior view of eye. **K**, **L**: GFP+ cells are seen at the posterior-dorsal aspect of the optic cup (bracketed by green arrowheads). Curved line indicates a tear in the RPE. **L**: Slightly higher magnification of **K**. **M**-**O**: Sections through eye in **K**. GFP+ cells are only found in the posterior retinal domain (green arrowheads). The ora serrate/CMZ, defined as the junction between the ciliary body (Collagen IX signal, **M**) and neural retina (Visinin signal, **N**), is indicated by the red arrow. GFP+ cells are not found at the CMZ. Asterisk indicates OCL. Note, isolated eye tissue is delicate and does not adhere well upon sectioning. In panel N the tissue has flipped during processing (white line). Boxed are in N shown at higher magnification in **O**. Scale Bars: 100 µm. N; Nasal, T; temporal, L; Lens.

We then labeled back from the OCL with the virus, mimicking the labeling with DiI (Figure 1E). Unlike OCL labeling, this required injection into the optic cup. Low-level infection was accomplished by diluting the virus stock and injecting the smallest volume, as monitored by the added contrast dye [32]. After reincubating for 3 days, embryos were removed and visualized. GFP+ cells were found in a discrete patch at a distance from the OCL (n=13), and were not arranged in a spoke-like line (compare Figure 3B with Figure 2A). Section analysis revealed GFP+ cells in the peripheral retina, but not in any of the intervening tissue between the Hu+ retina domain and the OCL (Figure 3D,E). Very specifically, no labeled cells were found at the junction between the ciliary body domain and the neural retina domain (red arrows, Figure 3D,E).

An advantage of retroviral labeling over DiI labeling is that the embryos can be followed over longer intervals, making it possible to determine the fate of labeled cells at advanced developmental stages. Embryos were reincubated for 8 days after labeling back from the optic cup (n=6), and in a wholemount view, no GFP+ cells were found; this was not surprising, as only the anterior portion of the eye was visible (Figure 3G,J). Once the eyes were dissected free from the head, and the majority of periocular mesenchyme and muscle removed, it was possible to see GFP+ cells constrained to the posterior/dorsal region of the optic cup (Figure 3H-L). Labeled cells were found in the neural retina associated with Visinin, a photoreceptor marker (Figure 3M,O). In some examples, embryos also had labeled cells in the RPE and overlying mesenchyme (not shown). As the label in these two tissues was always in the vicinity of the neural retina label and was similarly discrete, we viewed it as an indication of the site of injection. In contrast, the iris, ciliary body, ora serrata, and peripheral retina were all devoid of labeled cells (Figure 3N; red arrow denotes ora serrata). Interestingly, the labeled portion of the neural retina was displaced posteriorly compared to the relative position of the label at E5 (compare Figure 3D,E to Figure 3M,N).

### The optic cup lip is molecularly defined as a non-neural tissue

**Figure 4:**
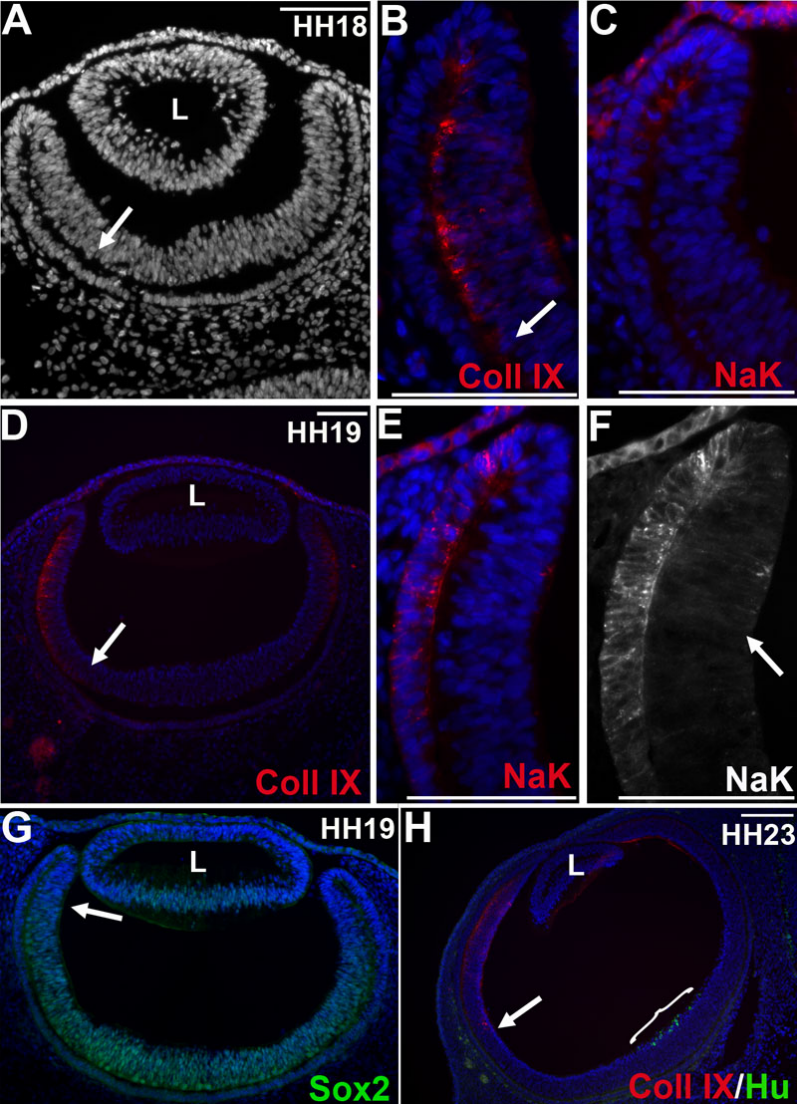
The early optic cup lip expresses mature fate markers. **A**-**F**: Transverse sections through eyes of 31 somite (**A**-**C**) and 38 somite (**D**-**G**) embryos labeled with Collagen IX (**A**, **B**, **D**) or NaK-ATPase (**C**, **E**, **F**). **A**: A low magnification of the section shown in **B**. **B**, **D**: Collagen IX (red) is present in the inner layer of the anterior optic cup. The arrows mark the posterior limit of Collagen IX expression. **C**, **E**, **F**: NaK-ATPase is present in the pigmented epithelium and the OCL. **F**: Expression of NaK-ATPase extends into the anterior of the non-pigmented, inner layer in a 38 somite embryo (arrow). **G**: Sox2 expression is present in the inner layer of the optic cup. Arrow marks the anterior limit of Sox2 expression. **H**: Transverse section through the ventral optic cup of a HH23 embryo. Hu is expressed at the posterior neural retina (bracket). The posterior limit of Collagen expression in the anterior eye is indicated (arrow). Scale Bars: 100 µm. HH; Hamburger Hamilton Stage, Coll IX; Collagen IX, NaK; NaK-ATPase.

**Figure 5:**
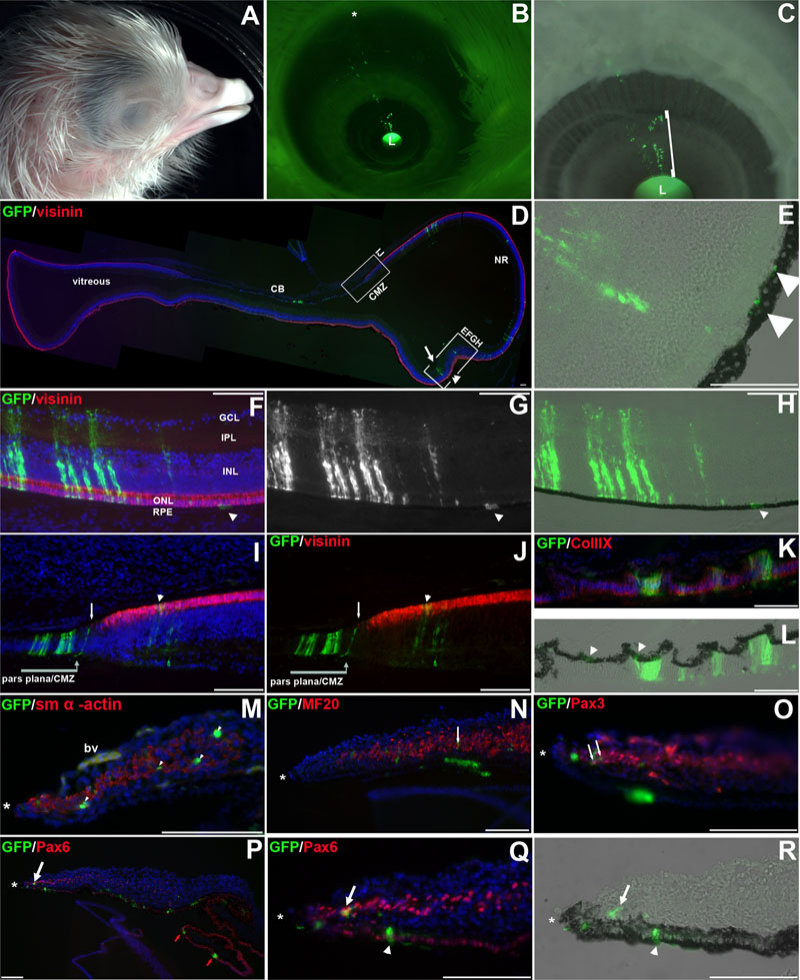
The OCL contributes cells throughout the extent of the eye. **A**-**C**: Wholemount view of an E17 embryo where the OCL was infected focally with GFP expressing replication-incompetent virus at E3. **A**: The embryo head before dissection to expose the eye. **B**: GFP expression in the eye of the embryo after removal of the eyelids and cornea. GFP can be seen from the OCL at the lens (L) to the cut edge of the eyelid (asterisk). **C**: Higher magnification view of **B**, showing retention of GFP expressing cells at the OCL. Bright cells have delaminated from the OCL into the overlying mesenchyme (bracket). **D**: Low magnification view of a section through the GFP expressing region of ciliary body and neural retina of the embryo shown in **A**-**C**. GFP is present in the ciliary folds and in the neural retina. The posterior extent of GFP expression is indicated (arrow). GFP is present in the RPE at a posterior position (double arrowhead). **E**: Higher magnification of the boxed area in **D**. GFP expressing cells in the RPE are indicated (double arrow). **F**-**R**: Adjacent sections through the embryo in **A**-**C**. **F**-**H**: GFP expressing cells are co-labeled with Visinin in the neural retina (**F**). **G**-**H**: GFP alone (**G**) and combined with the bright-field image (**H**). GFP is present in the inner neural retina layer and the overlying pigmented epithelium (arrow). **I**-**J**: GFP and Visinin at the CMZ. GFP is present in the pars plana of the ciliary body, the CMZ (arrow), and in photoreceptors at the peripheral neural retina (arrowhead). **K**-**L**: GFP is co-localized with Collagen IX expressing tissue in the inner layer of the ciliary folds and is also present in the outer pigmented ciliary epithelium (arrowhead; **L**). **M**: GFP is present in the smooth-muscle in the mesenchyme of the iris (arrowheads). **N**: GFP is present in the iris mesenchyme in an MF20 expressing myocyte (arrow) **O**: GFP is colocalized with Pax3 expressing cells in the iris mesenchyme (arrows). **P**-**R**: GFP and Pax6 colocalize (arrow) in cells delaminating from the OCL into the mesenchyme overlying the iris epithelium and in the epithelial layers of the iris (white arrowhead) and ciliary folds (red arrows). **Q**: A higher magnification of **P**. **R**: Corresponding bright-field image with GFP expression. Scale Bars: 100 µm. The anterior eye is to the left in all sections except **B** and **D**. *; OCL, L; lens, CB; ciliary body, CMZ; ciliary margin zone, NR; neural retina, PE; pigmented epithelium, ONL; outer nuclear layer, INL; inner nuclear layer, IPL; inner plexiform layer, GCL; ganglion cell layer.

It was not expected that cells labeled at the OCL would give rise to cells that populated the neural retina. Several molecular expression patterns within the newly formed optic cup have been interpreted to indicate that the OCL and the anterior portion of the optic cup represent presumptive ciliary body and iris tissue [11,14,33,34]. To characterize the OCL at the time of initial labeling (Hamburger-Hamilton [HH] stage 18 and 19), we analyzed the expression of Collagen IX and NaK-ATPase to mark the presumptive ciliary body and RPE, respectively (Figure 4). Collagen IX is expressed in the mature ciliary body, and as such, is used to define anterior eye tissues during optic cup development [10,35]. Conversely, before the accumulation of pigment granules, the RPE can be distinguished through its high expression of NaK-ATPase in the newly formed chick optic cup [36-38]. Collagen IX was evident in the inner layer of the optic cup at (HH stage 18/31 somites (Figure 4B), with intensity increasing at HH stage 19/38 somites (Figure 4D). Although the staining is faint at these early stage, intensity of signal increases with age, until it is very apparent that the entire inner layer of the anterior optic cup is expressing Collagen IX (data not shown). NaK-ATPase was present in the OCL and distal RPE at HH stage 18 (Figure 4C), increasing at HH stage 19 (Figure 4E,F) through more of the RPE and extending into the inner layer of the optic cup (Figure 4F; arrow). These stages of eye development were the same used in the OCL labeling experiments shown in Figure 1, Figure 2, and Figure 5, specifically HH stage 18 in embryos with 31 (Figure 2B) and 33 (Figure 2A,C) somites. At these stages, overt differentiation of the neural retina is not evident and only the most posterior portion of the neural retina expresses Hu one day later at E4 (Figure 4H). However, the preneurogenic portion of the optic cup can be identified by *Sox2* expression [14,39], which is expressed in the peripheral portion of the neural retina but does not extend to the OCL (Figure 4G). At the time of labeling, therefore, the anterior optic cup expresses anterior eye markers and pigmentation has been initiated. Expression of these markers are consistent with the usual interpretation that the OCL at this stage should be molecularly defined as nonneurogenic and thus not expected to form the neural retina. The findings presented here, in contrast, show that it is neurogenic.

### The optic cup lip is a progenitor pool throughout eye morphogenesis

Anterior-derived morphogenesis in the chick eye continues for over two weeks of development (Figure 5 and [40]). To assess the output of the OCL throughout eye morphogenesis, OCL cells were focally infected and embryos reincubated until E17 (Figure 5A). After removal of the eyelids and cornea, GFP expression was visualized directly (Figure 5B,C). GFP-expressing cells remained in the OCL, adjacent to the lens, and extended as a thin spoke-like arrayed population toward the back of the eye. The tagged cells were present throughout the iris and could be detected to the point where the eyelid had been cut (Figure 5B; asterisk). As at younger collection times, there was little expansion of GFP expression around the pupillary circumference, suggesting that growth persists in a linear and ordered anterior (at the lens) to posterior (at the optic nerve) manner.

### The optic cup lip’s contribution to the neural retina

The OCL’s contribution to the neural retina observed at E5 was unexpected, as it is assumed that the OCL is specified as presumptive iris and ciliary body [14,41,42]. To determine the extent of OCL-derived neural retina, sections through the plane of the GFP spoke-line of E17 eyes were examined. GFP-expressing cells were present in multiple tissues of the eye (Figure 5D-R). At low magnification, a section through the ciliary body and neural retina highlights the extent of OCL contribution to the neural retina (Figure 5D; arrow). GFP expression in a more central region of the neural retina was additionally associated with GFP in the adjacent overlying RPE (arrowheads; Figure 5DE). This confirms that at time of labeling, optic cup invagination was complete, and OCL-driven growth was adding progeny to both the inner and outer layers of the optic cup. GFP expression was present throughout the mature neural retina. GFP was expressed in the RPE (Figure 5D-H; arrows), in association with Visinin-expressing photoreceptors in the outer nuclear layer (Figure 5F; green label), in neurons in the inner nuclear layer, in axons traveling through the inner plexiform layer, and associated with retinal ganglion cells in the inner ganglion cell layer of the retina (Figure 5F).

GFP-expressing cells were evenly distributed across the boundary between the neural retina and the pars plana of the ciliary body (Figure 5I,J; arrows). This morphologically distinct region, the ora serrata, is analogous to the stem cell–containing ciliary marginal zone (CMZ, also referred to as the circumferential margin zone) of lower vertebrates [9,43-45].

### Multiple cell fates in the anterior eye originate from the optic cup lip

The optic cup derivatives in the anterior eye form the epithelial portions of the ciliary body and iris. Neural crest–derived mesenchymal cells associate with these epithelia to create the highly vascularized ciliary body stroma and a portion of the pupillary sphincter muscle, respectively. To determine the extent of OCL-derived cells through the various anterior tissues, sections through GFP-expressing spoke lines obtained after E3 OCL infection were labeled with cell fate markers. GFP expression was present in the pars plana of the ciliary body, localized both in the Collagen IX–expressing inner nonpigmented layer (Figure 5K,L) and in the overlying pigmented ciliary epithelium (Figure 5K,L; arrowheads). GFP was also present in the more anterior ciliary folds (Figure 5P; red arrows).

The iris is the anterior limit of the optic neuroepithelium (Figure 5C; open bracket). Embryos examined at E17 showed that GFP-expressing cells were present from the anterior to posterior extent of the iris (Figure 5B,C). Sections confirmed GFP expression in the iris epithelium (Figure 5P-R; arrowheads) and in the mesenchyme overlying the iris (Figure 5M-Q). In both avian and mammalian species, the smooth muscle component of iris musculature delaminates from the optic epithelium. Avian iris muscle also includes skeletal muscle, a part of which is derived from the neuroepithelium. In the iris mesenchyme, GFP colocalizes with Pax6 (indicative of optic epithelial origin), Pax3, smooth muscle alpha-actin, and myosin (Figure 5M-Q).

### Iris differentiation confirms a persistent anterior-posterior maturation gradient during eye development

**Figure 6:**
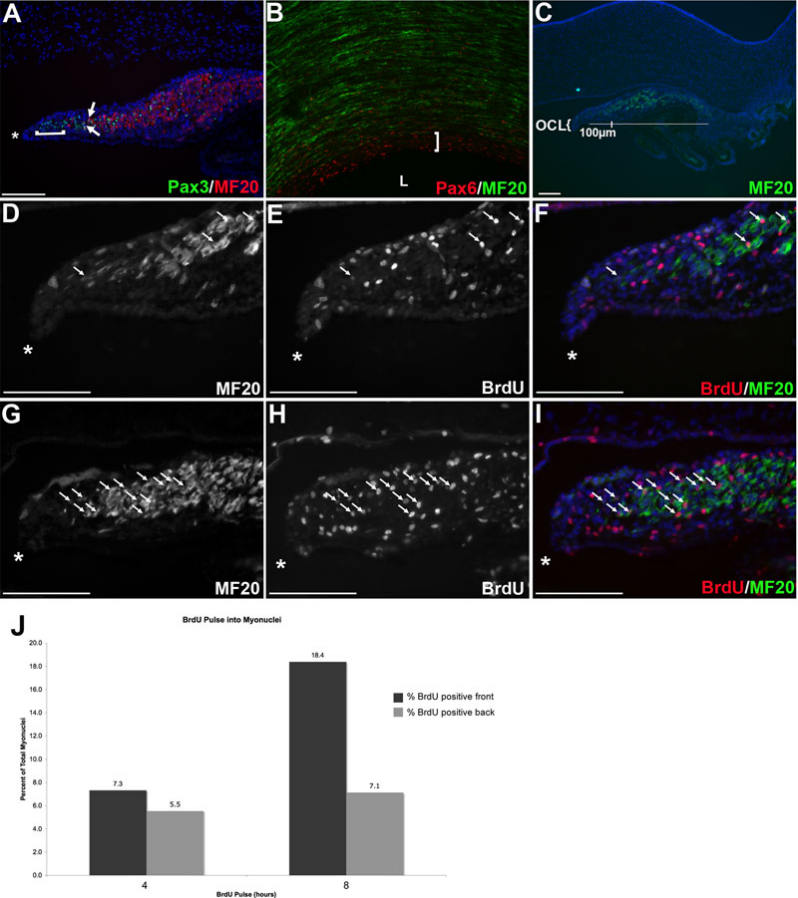
An anterior-posterior maturation gradient is maintained through development of the iris from the OCL. **A**, **C**-**I**: Transverse sections through E14 eyes. **A**: A section labeled with Pax3 and MF20. MF20 is absent from the mesenchyme closest to the OCL (bracket). Pax3/MF20 are expressed in the same cells at the anterior position in the skeletal muscle (arrows). **B**: A confocal image of a wholemount E16 eye labeled with Pax6 and MF20. Bracket indicates a Pax only zone in the eye anterior to musculature. **C**: A section labeled with MF20. The bar indicates the iris sphincter muscle and the anterior most 100 µm. **D**-**I**: Sections through the eyes of embryos exposed to BrdU for 4 h (**D**-**F**) or 8 h (**G**-**I**) before harvesting. Skeletal muscle is labeled with MF20 (**D**, **F**, **G**, **I**) and arrows indicate myonuclei that have incorporated BrdU (**E**, **F**, **H**, **I**). **J**: Graphic summary of BrdU pulse data. There is a significant difference in the percent of nuclei that have incorporated BrdU comparing the anterior and posterior muscle after an 8 h (p<0.01) but not 4 h (p>0.05) pulse. Scale Bars: 100 µm, * indicates anterior of cup/anterior of pupillary margin). L; lens, OCL; Optic Cup Lip.

An anterior-posterior differentiation gradient during retinal development is well documented [46-51]. Iris muscle development was used to confirm that this gradient was maintained throughout the development of a late differentiating tissue. We used the expression of Pax3/6 and a differentiated muscle protein to examine a maturation gradient in the iris muscle. Pax6 expression marks optic neuroepithelium–derived cells and Pax3 is a marker of myogenic precursors. It was found that Pax3 colocalized with Pax6 in delaminating iris cells (not shown). A younger, Pax-only zone (Figure 6A,B; brackets) persisted at the front of the eye from the onset of overt muscle differentiation (not shown) through to the mature stages (Figure 6). Posterior to this, Pax3 and MF20 were found together in a few cells (Figure 6A; arrows), and finally, a zone of differentiated myocytes with some Pax3/6 expressing cells interspersed confirmed the transition from less differentiated anterior tissue. In wholemount, a Pax6-only zone was evident adjacent to the lens (Figure 6B; bracket), with differentiated skeletal muscle posterior to this zone.

Terminally differentiated skeletal muscle is postmitotic; therefore, we used a BrdU pulse protocol to determine whether differentiating myocytes were preferentially added to the anterior muscle from mitotic progenitors (Figure 6C-J). Few myonuclei will incorporate BrdU and express myosin after a 4 h pulse, while there should be a significant increase in BrdU/myosin co-expression after an 8 h BrdU pulse. Quantification of the number of BrdU-positive nuclei contained in differentiated (myosin expressing) myocytes allows identification of sites of terminal myogenic differentiation, and can therefore be used to assess the maturation status of skeletal muscle [25,52,53]. E14 embryos were exposed to BrdU for 4 or 8 h and the number of myonuclei and BrdU-positive myonuclei were counted in sections through the eyes (Figure 6D-I; asterisks indicate most anterior aspect of optic cup, the pupillary margin). After 4 h of BrdU exposure, there was no significant difference in the number of BrdU-labeled myonuclei between the anterior and posterior of the muscle (p>0.05). In contrast, there was a significant increase in the number of BrdU-labeled myonuclei in the anterior 100 µm, compared with the posterior, after 8 h (p<0.01). These data confirm a preferential addition of myoblasts to the anterior aspect of the muscle, and shows that eye morphogenesis continues with an anterior-posterior maturation gradient through differentiation of the adult structure.

Together, these data highlight that the neural retina and tissues of the anterior eye share a common origin in the OCL of younger embryos and do not represent distinct embryonic eye compartments. Further, the OCL has a sustained role as a progenitor pool throughout eye morphogenesis, contributing cells to the anterior aspect of the eye through mitotic activity, resulting in a persistent anterior to posterior maturation gradient.

## Discussion

This study represents the first lineage analysis to determine the developmental relationships between discrete eye tissues in higher vertebrates. We have directly examined the derivation of mature eye tissues from the newly formed OCL, and report that with the exception of the posterior neural retina and associated posterior RPE, the OCL is a common stem/progenitor pool for optic epithelia and for the cells that delaminate from the OCL to contribute to the musculature of the iris at the endpoint of eye development.

### The ciliary margin zone is populated with progeny from the optic cup lip

The CMZ is the proliferative zone at the far peripheral edge of the neural retina, where the ciliary epithelium begins. It is a well defined adult stem cell niche in amphibians and fish, and similar activity has been found in juvenile chickens [43,54,55]. In amphibians and fish, adult eye growth is driven by proliferation from the progenitor pool at the CMZ [44,45] but in higher vertebrates, the CMZ is a late embryonic structure and very little retinal expansion is attributed to CMZ-derived progenitors ([40] reviewed in [47,56]). Whether and where a retinal stem cell population might be found in the mature eye of higher vertebrates remains in question. In this report, we identified a novel pool of stem/progenitors for the entire optic neuroepithelium, including the potential retinal stem cells in the chick CMZ. It was clear that labeled OCL progenitors were incorporated into the CMZ, a structure that was clearly identifiable by E10 (Figure 5), and that the label in the CMZ was part of a linear progression of labeling, from neural retina to iris tissue. All efforts to label the CMZ alone (Figure 4), without concomitant OCL labeling, were unsuccessful; this supports the lineage relationship between the two structures. The results from this study indicate that the OCL contains pluripotent cells during embryogenesis; they highlight the need for further study into the maintenance of these cells during embryogenesis, as well as whether this potential is retained in adulthood.

### The optic cup lip contributes significantly to the neural retina during development

**Figure 7:**
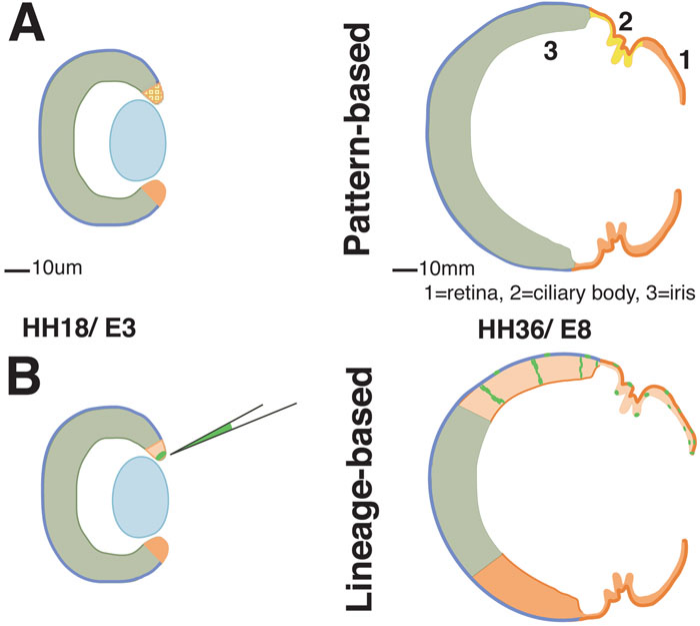
The optic cup lip is a common progenitor pool for retina and anterior eye. **A**: Model of optic cup subdivision into retinal and anterior compartments, based upon gene expression patterns and proliferation indices. The early optic cup would consist of an expansive retinal compartment (green) and separate anterior eye compartment (orange), situated at the peripheral optic cup. With development, the retina would grow through intrinsic expansion and the anterior structures, ciliary body and iris, would be exclusively derived from the optic cup lip. **B**: Permanent tagging of OCL cells reveals a substantial contribution from the OCL to the retina in addition to the anterior eye tissues. Intrinsic expansion of the inner layer of the early optic cup accounts for only a portion of retinal growth.

Models of eye development are based largely on amphibian lineage analysis, short-term studies of optic vesicle labeling, gene expression patterns, and proliferation studies in many species. In higher vertebrates, these data support models in which the neural retina is derived exclusively from the distal optic vesicle, such that after invagination, the distal portion of the vesicle becomes the most posterior neural retina (Figure 7A) [15,42,57-60]. During development, the retinal component of the nascent optic cup would then expand through intrinsic proliferation, while continued addition of extrinsically derived retinal progenitors at the periphery of the neural retina is proposed to vary from little to none depending on the species studied ([32,46,47,49,51,61] and reviewed in [11]). Other research favors a model in which a CMZ-like population, located at the junction between neural and non-neural tissue and physically separate from the OCL, drives the developmental growth of the eye [19,20,62,63]. In no cases is it considered that the very tip of the optic cup is involved in retinal growth. At the early stages of frog eye development, before the CMZ is clearly separable from the OCL at the cup margin, precursors can be labeled that give rise to both neural retina and RPE, similar to the direct lineage analysis presented here [64]. In addition, this report follows those lineage relationships, for the first time, through the duration of eye development, and demonstrates that the precursor cells at the OCL contribute significantly to all tissue derived from optic epithelia (Figure 2 and Figure 5).

### The optic cup lip as a specialized niche

The OCL, as the most anterior portion of the optic cup, is often described as being specified early for differentiation into iris and ciliary body tissue [14,41,42]. In the newly formed eye, the anterior portion of the optic cup expresses several markers that persist until overt ciliary body histogenesis, including Collagen IX, a vitreal protein that is secreted from the ciliary body; this can be considered a marker of functional ciliary tissue [65]. We show here that the neural retina is derived from OCL cells, despite the lip having been targeted after the molecular segregation of the optic cup into neural (Sox2) and nonneural (Collagen IX) domains (Figure 4). Collagen IX–expressing portions of the anterior optic cup, which include the OCL, can also recreate the neural retina in a chick model of retinal regeneration [63]. Collagen IX may not define a terminal fate assignment; a similar lack of congruence between marker and fate has been seen before [66]. Alternatively, the OCL may have a mixture of defined and undefined cells; however, Sox2 expression is not conversely seen at the OCL (Figure 4 and [14]). These observations suggest that the boundary between neural and nonneural tissues in the anterior optic cup is flexible during development. This flexible boundary might still be present in adult tissue. There is some evidence for retinal progenitors in the ciliary epithelium (anterior to the CMZ) [67,68], and retinal markers have been found to persist in the pars plana of the ciliary body in primates [6]. The eye may be particularly fluid in its terminal differentiation; for example, it is well documented that the differentiated and pigmented outer layer of the optic cup can be induced to dedifferentiate and switch fates to form neural retinal tissue [62].

Tagging slightly posterior to the OCL, with both DiI and retrovirus, produces isolated patches of labeled cells only in the neural retina, similar to findings reported by Fekete et al. [32]. These patches are found more posteriorly when observed at later time points (Figure 3; E5 compared to E10). At the time of labeling, the targeted cells would be defined as presumptive retina, based on known expression patterns (Figure 4); they are the cells at the border between the ciliary body (expressing Collagen IX) and the specified neural retina (as seen by Sox2 staining at nascent optic cup stages). However, they do not populate the mature CMZ, as seen at E10 (Figure 3). Only tagging the OCL results in labeled cells throughout all the tissues of the eye, a finding that lends support to our conclusion that the OCL is a specialized niche.

### Cells in the optic cup lip are multipotent progenitors

This study cannot definitively prove that a single OCL cell produces progeny that contributes to all neuroepithelial derivatives; the spoke-like arrays have not been systematically assessed for their viral insertion points, and the infections were not performed at strict single virion concentrations. It is possible that a tagged population would contain subsets of specified progenitors in very close proximity, and this could explain how the retina, CMZ, RPE, iris epithelium, ciliary epithelium, and pupillary muscle would all contain labeled cells. Currently, we prefer a model in which a given OCL cell is multipotent and gives rise to all these differentiated types (Figure 7B). The following lines of evidence support this interpretation:

1. In labeled eyes, over 90% of GFP+ embryos retained their label in the OCL (n=33), particularly those examined at E17;
2. Alternative patterns, where only the retina or only the ciliary body were labeled, were not observed;
3. The low percentage of targeted embryos that were infected (5%) is supportive of low virion introduction;
4. The number of labeled cells retained at the OCL in E6 specimens was never observably greater than the number of labeled OCL cells in E17 specimens, supporting self-renewal; and
5. Label in the neural retina coincided with label in the neighboring RPE, indicating emergence of these cells from a common pool in the OCL.

Apart from this report, several other studies have presented results that support an interpretation that the OCL is a progenitor center for eye growth. Coulombre and Coulombre [69] classically demonstrated that the chick retina can reform after complete removal. The description of the surgery makes it clear that the retina was removed by cutting at the hinge area, likely leaving the OCL intact. (Note that regrowth required added factors, supplied by ectopic tissues.) More recently, Spence et al. [63,70] expanded on these experiments. Using DiI labeling, they also observed that the regenerated retina expanded in a linear fashion from the hinge of the optic cup. Finally, in vitro experiments by Wilbold and Layer [50] showed that proliferative multipotent progenitors were found in cultures of chick retina that included the OCL populations, as compared to the CMZ populations. These reports are conclusive evidence of the presence of neural retina precursors in the very anterior margin/OCL of the eye and are explained by the definitive lineage analysis presented in this study. In addition, we show that the RPE population is also derived from the OCL, which was not examined in these prior reports. We were also uniquely able to follow the lineage into a fully differentiated eye, to find that the OCL progenitors also give rise to smooth muscle. The range of different cell types produced is experimentally provocative: Future work will address the question of how the OCL creates this range and whether a small population of stem cell remain at the OCL, or whether they are depleted as a mechanism of producing diversity.

These studies were all performed in the chick embryo, and may be specific for avians. It is not currently possible to accomplish long-term, direct lineage analysis in the mammalian eye. However, there are intriguing hints that the OCL may contribute to the neural retina in mice. Site-specific somatic recombination with the tyrosinase-related protein 1 promoter (Trp1) illustrates a lineage relationship between cells at the OCL and neural retina. Trp1, Trp2, and tyrosinase are involved in the melanin-synthesis pathway and are highly expressed in the developing RPE before the onset of pigmentation [71]. Although considered an RPE gene, the expression of Trp1 itself, and marker genes driven by the Trp1 promoter, extend around the hinge in the anterior optic cup, with a small domain of expression at the tip of the inner layer [71,72], similar to Trp2 [72,73], and as we show with NaK-ATPase expression (Figure 3). When combined with LacZ or Z/AP reporter mouse lines, the Trp1 promoter–driven Cre displays the expected expression in the RPE and at the tip, and shows a developmentally expanding domain of reporter gene expression in the inner layer of the optic cup, including the ciliary body, CMZ, and peripheral neural retina. Mori et al. [71] hypothesized that the expression domain, which did not overlap with any ectopic Cre expression, was potentially indicative of neural retina progenitors being present at the lip of the optic cup. Their data support a model of incremental growth of the peripheral neural retina and anterior structures from a shared progenitor, as we have demonstrated directly with lineage analysis. Whether the morphogenetic parameters demonstrated here for the avian eye can be directly transferred to the mouse remains open to testing.

#### Conclusion

Testing the potential of the optic cup during eye development shows that, in birds, the OCL is a specialized progenitor niche containing pluripotent cells. OCL cells populate the anterior eye, including a large portion of anterior neural retina and RPE (Figure 6B). This favors a model of eye morphogenesis in which the majority of the neural retina is derived progressively from the OCL, rather than the prevailing model in mammals and birds where the neural retina is derived as a single unit from the distal optic vesicle upon its invagination to form the optic cup. This model promotes a late separation of neural and anterior eye fates highlighting the requirement for further studies of fate plasticity during eye development.
